# Therapeutic efficacy of Transpedicular Intracorporeal cement augmentation with short segmental posterior instrumentation in treating osteonecrosis of the vertebral body: a retrospective case series with a minimum 5-year follow-up

**DOI:** 10.1186/s12891-019-2671-4

**Published:** 2019-06-29

**Authors:** Hongli Deng, Yibing Li, Jinsong Zhou, Xiaodong Wang, Jinpeng Du, Wenjie Gao, Dingjun Hao

**Affiliations:** Department of Spine Surgery, Xi’an Honghui Hospital, Xi’an Jiaotong University, Xi’an, China

**Keywords:** Transpedicular intracorporeal cement augmentation, Short segmental posterior instrumentation, Osteonecrosis of the vertebral body

## Abstract

**Background:**

Transpedicular intracorporeal cement augmentation (TCA) with short segmental posterior instrumentation (SSPI), which provides an ideal immediate analgesic effect and long-term reconstructive stability, is thought to be a sensible advancement to the operative strategy in treating osteonecrosis of the vertebral body (ONV). However, long-term follow-up studies about the treatment are scarce.

**Methods:**

Forty-six ONV patients (22 males and 24 females, mean age of 62.8 ± 7.11 years) underwent TCA with SSPI were retrospectively analyzed. During follow-up, clinical outcomes, such as the Visual Analogue Scale (VAS) score and the Oswestry Disability Index (ODI) score, were evaluated, as well as radiologic outcomes, such as the average vertebral height and kyphotic angle.

**Results:**

A total of 36 patients completed a follow-up period of at least 5 years (mean follow-up period of 67 ± 4.2 months). Among them, seven patients experienced complications, i.e., pneumonia (2/36, 5.56%), screw loosening (2/36, 5.56%), moderate hematoma in the subcutaneous tissue (1/36, 2.78%), and cement leakage (2/36, 5.56%). Compared to the preoperative score, the mean VAS score was significantly reduced 6 months postoperatively (*P* < 0.05), and it concluded being virtually identical to the preoperative score (*P* > 0.05). The mean ODI score exhibited a comparable trend. Regarding the radiologic evaluation, the mean kyphotic angle and average vertebral body height were significantly corrected postoperatively (both *P* < 0.05). However, these radiological parameters were maximally ameliorated during the direct postoperative period and slowly deteriorated over time.

**Conclusion:**

The present study shows that TCA with SSPI may be only mildly effective for symptom relief and correction of kyphotic deformity during a relatively long follow-up, thus we do not recommend it for ONV.

## Background

Osteonecrosis of the vertebral body (ONV), also known as Kummell’s disease, which was first described by Hermann Kummell in 1891, is a delayed complication of osteoporotic vertebral compression fractures (OVFs) [1, 2]. Although most OVFs can be treated conservatively, approximately 7–24% develop into ONV [3, 4]. Though many studies of ONV have been conducted, the exact pathology and pathogenesis are still unclear. Most researchers agree that vascular injury of the vertebral body leads to impaired fracture healing [4–6]. Mild back pain appears after trivial trauma, and after an asymptomatic period of weeks to months the pain may recur or even become worse. Sometimes patients suffer from significant kyphotic deformity and neurologic involvement [5, 7]. Since conservative treatments, including analgesics and bed rest, bring little benefit in pain relief, surgical interventions of ONV may be the first treatment choice [8, 9]. Percutaneous kyphoplasty (PKP) and percutaneous vertebroplasty (PVP) have been suggested as effective and minimally invasive procedures for stage I and II ONV [1, 10–12]. However, PVP and PKP technologies possess the intrinsic defect of cement leakage and a concomitant reduction of the correction angle. The vertebral height loss after PVP or PKP is thought to be associated with segmental instability and insufficient interaction between bone and cement [13–15]. Short segmental posterior instrumentation (SSPI) associated with transpedicular intracorporeal cement augmentation (TCA) is considered to be a reasonable improvement in the operative approach as it offers an ideal immediate analgesic effect and long-term reconstructive stability. However, long-term clinical studies about this treatment are scarce.

### Rationale for the present study

Limited and contradictive studies have addressed ONV management, thus our goal was to evaluate the outcomes of TCA with SSPI for treating ONV during a relatively long follow-up period (> 5 years).

## Methods

### Patient characteristics

This study was approved by the Ethics Committee of Honghui Hospital (No. XAHHLLSP1022), and written consent was obtained from all of the patients. A total of 46 patients with ONV who were treated by the method of TCA with SSPI between July 2010 and July 2012 were enrolled in the present study, i.e., 22 males and 24 females, with a mean age of 62.8 ± 7.11 years. Each patient had only one affected vertebral body. The vertebral fractures were as follows: T11, 3 patients (6.5%); T12, 10 patients (21.7%); L1, 12 patients (26.1%); L2, 14 patients (30.4%); L3, 1 patient (2.2%); L4, 4 patients (8.7%); and L5, 2 patients (4.3%). All these patients had a vacuum cleft of the vertebral body that was verified by preoperative plain radiographs and computed tomography (CT) scanning. The accumulation of air or liquid was verified via MRI. Two radiologists assessed the imaging examinations (the plain radiographs, CT, and MRI), and only when both agreed with the diagnosis of ONV was included in the study.

### Inclusion and exclusion criteria

Inclusion criteria were the following: (1) patients were assessed by physical evaluations, plain radiographs, CT, and MRI; (2) definitive determinations of an intravertebral cleft, vacuum phenomenon, and gas or liquid area in fractured vertebrae showed by radiographs, CT scans or MRI; (3) patients with symptoms or signs consistent with the ONV lesion, including tenderness in the lesion area and back pain during flexion and extension; and (4) older patients with a T score under − 3.0. The indication for treatment was the absence of pain relief during the first 4 weeks following conventional treatment. The fractured vertebra with intravertebral fluid or air pockets was chosen and the area was paired with the region of pain to establish which spinal segment required treatment.

Exclusion criteria were the following: (1) history of stroke, diabetes, rheumatoid disease, dementia, significant medical condition necessitating rigorous treatment, or simple osteoporotic compression fractures without ONV; (2) multiple vertebral fractures; (3) prior vertebral fractures in the same or a nearby vertebra above or beneath the fractured vertebra and prior spinal operation and vertebroplasty; and (4) patients with neurological compromise.

### Surgical technique

**Fig. 1:**
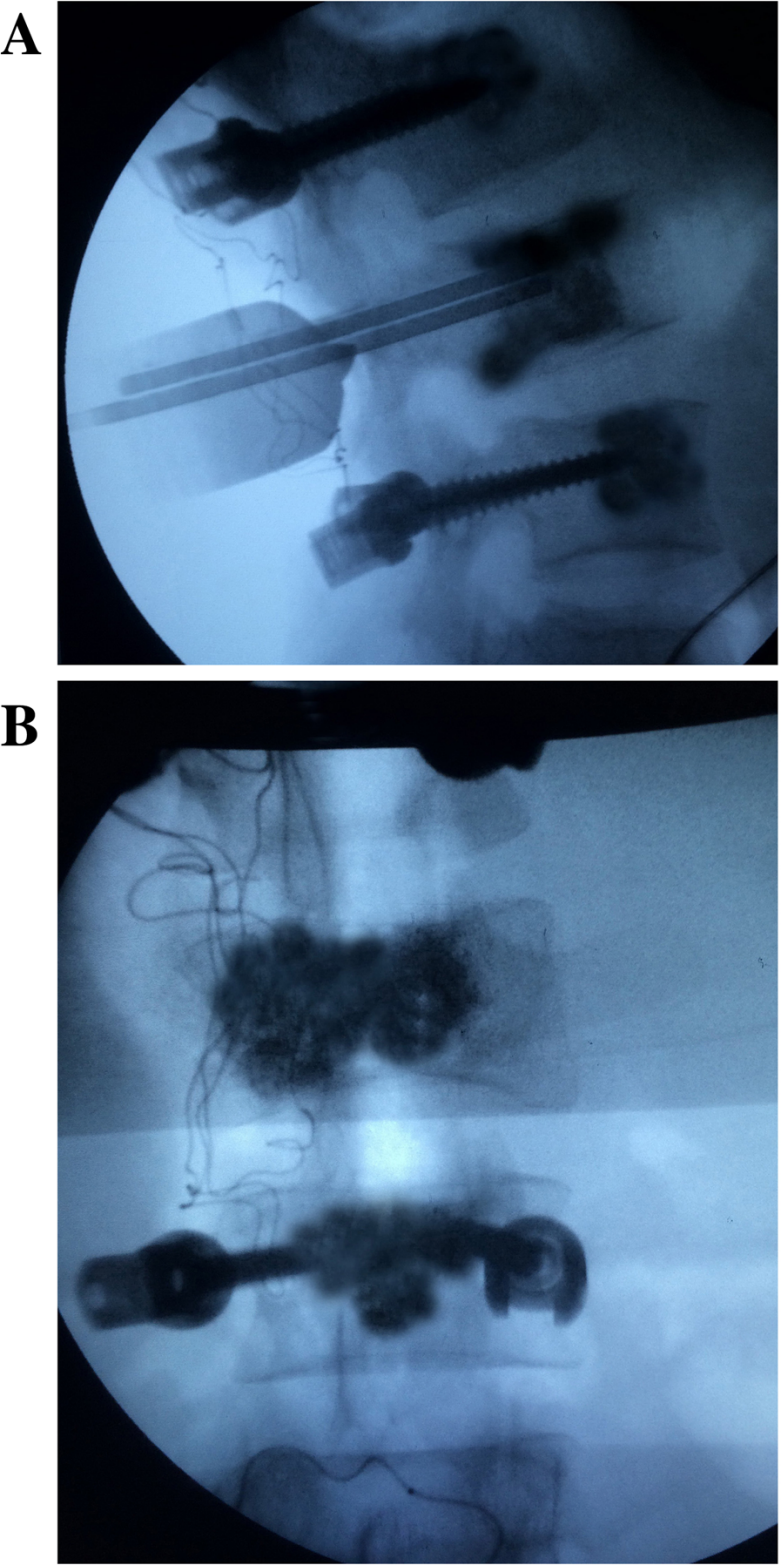
Interoperative fluoroscopy demonstrates dual tunnels placed in the correspondent target area. One is inserted into the cleft and the other one is underneath the cleft

Operations were conducted under general anesthesia. Patients were placed in the prone position with sponge mats bracing the upper chest and pelvis to allow maximum extension of the spinal column. A typical posterior midline strategy was utilized to reveal the posterior portion of the spine for surgical tools, including one level over and under the buckled vertebra. Hollow cement pedicle screws were implanted into the bilateral pedicles of the proximal and distal nearby vertebrae. The injection volume of polymethylmethacrylate (PMMA) cement was 1–1.5 ml per screw. For the diseased vertebra, two tunnels were placed under fluoroscopy, with one in the cleft and the other beneath the cleft (Fig. 1). Cement was injected slowly into the diseased vertebra under continuous fluoroscopy in the lateral plane. Cement injection was ceased when the cement completely filled the cleft, and the region underneath the cleft was filled with cement over 50% of the transverse diameter of the sabotaged vertebral body. The cement volume was approximately 4.5–8 ml. After the cement injection was completed, ordinary pedicle screws (35 mm in length and a diameter of 6.5 mm) were put into place. The rods were then put into place and secured after a reduction of the cement temperature. Reduction was not performed, and lamina and facet joint fusion was not implemented. After attaching the rods to the screws, the surgical opening was closed in layers. A drain was routinely inserted into the deep wound space. After the drain was removed, the patient was allowed to ambulate. Antiosteoporotic treatment was a regular option for the patients. All the patients underwent postoperative physical therapy for 6 months. Three months of total contact brace application was recommended.

### Data collection and analysis

Demographic, clinical, and radiologic data were gathered from medical records. Following surgery, an evaluation was conducted at established intervals: right after the operation, at 3 months, at 6 months, at 1 year, and every year thereafter. The impact on the patient’s daily life was assessed using the Oswestry Disability Index (ODI). The Visual Analogue Scale (VAS) score was used to assess the degree of back pain. Anteroposterior and lateral radiography were conducted at every follow-up visit. Transformation of the vertebral body was analyzed through serial follow-up plain radiographs. Vertebral height was quantified as height in millimeters on the vertebral borders at the anterior and posterior margins of the sabotaged vertebral body. The average vertebral body height was determined as the mean of the anterior and posterior body height. The kyphotic angle was established with Cobb’s method on a standard lateral radiograph. Each of the heights and angles was measured with the picture archiving and communication system (PACS) and the relevant computer software (PiViewSTAR 5.0, INFINITT, Seoul, Korea).

### Statistical analysis

Nonparametric statistical analysis was performed due to the relatively small sample size. Statistical analysis included the Fisher exact test, the Mann–Whitney U test, and the Wilcoxon matched pair signed-rank test. The correlation coefficients between the clinical results and imaging measurement was evaluated with the Spearman test. SPSS software version 19.0 (SPSS, Chicago, IL, USA) was used to conduct the statistical analysis. A two-tailed *P*-value of < 0.05 was considered to be statistically significant.

## Results

### Follow-up of patient characteristics

Six patients were lost to follow-up, one died due to myocardial infarction, and three had a vertebral body fracture, which left a total of 36 patients. Each of the patients had a follow-up period of at least 5 years, with a mean follow-up period of 67 ± 4.2 months. The average hospital stay was 10.4 ± 2.3 days, the average surgical time was 132 ± 15.5 min, and the average blood loss was 410 ± 30.5 ml.

### Surgical complications

Of the 36 patients, seven patients experienced complications, namely, pneumonia (two patients, 5.56%), screw loosening (two patients, 5.56%), moderate hematoma in the subcutaneous tissue (one patient, 2.78%), and cement leakage (two patients, 5.56%). No revisions were required in the patient with screw loosening because no progression was found during the long-term follow-up. Cement leakage was located at the paravertebral portion. No neurologic deficits were found to be associated with cement leakage. No revision was needed to deal with the leakage.

### Clinical results

The preoperative VAS score was 7.4 ± 1.6. At the 3-month postoperative appointment (3.6 ± 1.3), a significant reduction was found (*P* = 0.01). Succeeding VAS scores throughout the postoperative follow-up were 4.8 ± 1.4 at 12 months, 5.0 ± 1.3 at 2 years, and 5.1 ± 1.2 and 5.6 ± 1.1 at 3 and 5 years, respectively. The mean VAS scores at the 2-, 3-, and 5-year follow-up visits were significantly elevated compared to the mean VAS score at 3 months. When compared with the preoperative VAS score, no significant differences were found (Table 1). The mean ODI score was 72.4 ± 4.6 preoperatively, 32.4 ± 12.6 at 3 months postoperatively, 42.4 ± 6.6 and 48.4 ± 6.6 at 1 and 2 years, and 62.4 ± 8.6 at the last follow-up appointment. While the mean ODI score was significantly reduced until 1 year following surgery, this reduction was almost lost 5 years postoperatively. No statistically significant difference was found between the 2-, 3-, 4-, and 5–year follow-up ODI scores and the preoperative score (Table 1).

**Table 1:** Summarized Data of Clinical Outcomes

Time	VAS	*P*	ODI	*p*
preoperative	7.4 ± 1.6	...	72.4 ± 4.6	...
3 days postoperative	3.6 ± 1.3	0.02*	N/A	...
1 month postoperative	3.4 ± 1.4	0.02*	42.4 ± 8.9	0.03*
3 months postoperative	3.2 ± 1.2	0.01*	32.4 ± 12.6	0.01*
6 months postoperative	3.1 ± 0.9	0.01*	30.4 ± 6.6	0.001*
12 months postoperative	4.8 ± 1.4	0.08^△^	42.4 ± 6.6	0.04*
24 months postoperative	5.0 ± 1.3	0.14^△^	48.4 ± 6.6	0.13^△^
36 months postoperative	5.1 ± 1.2	0.16^△^	50.4 ± 4.9	0.23^△^
48 months postoperative	5.2 ± 1.7	0.21^△^	55.6 ± 7.6	0.33^△^
60 months postoperative	5.6 ± 1.1	0.31^△^	62.4 ± 8.6	0.49^△^

*p < 0.05, ^△^*p* > 0.05

### Radiologic findings

**Fig. 2:**
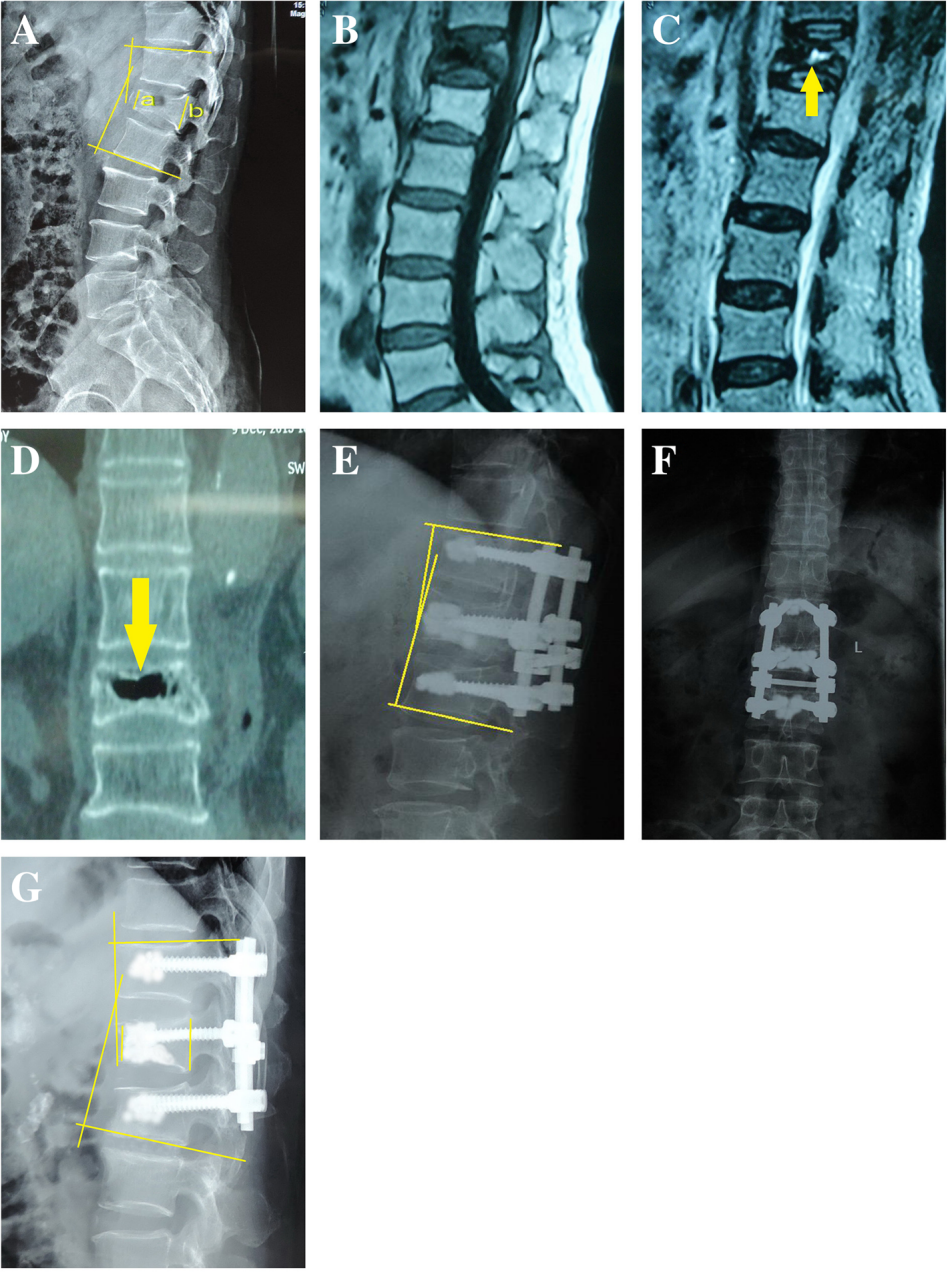
**a**: The measurement of kyphotic angle and average vertebral body height = (a + b) /2; **b**, **c**: local collection of fluid on MRI; **d**: sagittal CT reconstruction demonstrates the intervertebral cleft; **e**, **f**: at 3 days postoperatively, radiographs demonstrate correction of kyphosis and restoration of vertebral height; **g**: at 5 years postoperatively, radiographs demonstrate vertebral height loss in both the sabotaged and caudal vertebrae and an increase in kyphosis angle

Kyphosis angle had positive correlation with VAS score (Spearman r = 0.463, *P* <  0.0001) and ODI score (Spearman r = 0.490, *P* < 0.0001), but vertebral height was not found to be correlated with VAS score or ODI score (Both *P* > 0.05). The mean preoperative kyphotic angle was 34.3° ± 5.4. It was significantly reduced at the immediate postoperative follow-up visit (*P* = 0.001). There was also a significant difference between the preoperative mean vertebral height and 3 days postoperatively (*P* = 0.01). At 1 year postoperatively, 26 patients developed progressive vertebral height loss. The mean vertebral height was 15.2 ± 3.5 mm at the final follow-up visit. When compared with the preoperative height, no statistically significant difference was found (*P* = 0.73). The kyphotic angle displayed a similar change in accordance with vertebral height (Table 2). We observed caudal vertebral body height loss in 23 patients (Fig. 2).

**Table 2:** Summarized Data of Radiologic Findings

Time	Kyphotic Angle	*p*	Average vertebra height	*p*
Preoperative	34.3° ± 5.4	...	14.2 mm ± 3.9	...
3 days postoperative	8.4° ± 4.4	0.001*	20.9 mm ± 4.7	0.01*
12 months postoperative	14.3° ± 6.2	0.03*	16.8 mm ± 6.7	0.23^△^
36 months postoperative	16.3° ± 4.7	0.09^△^	16.2 mm ± 3.0	0.36^△^
60 months postoperative	28.6° ± 6.8	0.43^△^	15.2 mm ± 3.5	0.73^△^

*p < 0.05, ^△^*p* > 0.05

## Discussion

Based on the pathophysiology of ONV, the cleft in the vertebral body is difficult to heal. There is no typical treatment for spinal fragility connected with ONV. The treatment choices greatly rely on three factors: the pain intensity, the degree of the kyphotic malformation, and the existence of neurologic deficits. Conservative therapies include bed rest and brace placement; however, recent studies have favored surgical intervention [8, 9]. Surgical options primarily include PKP, PVP, and posterior instrumentation. Surgery can reduce the vertebral deformity, relieve symptoms, and shorten the time of immobilization. While anterior thoracolumbar surgeries, in which the diseased body is eliminated (corpectomy) and an interposition bone graft is stationed, have stringent technical requirements and are possibly hazardous in aged patients [16, 17]. PKP shows an excellent analgesic effect in patients with ONV; however, the long-term clinical effects of PKP in treating ONV are not ideal [13, 18–21]. In addition, a few recent evaluations detailed preexisting ONV as the greatest risk factor for recollapse of vertebrae previously treated with PVP [15, 22, 23].

It was hypothesized that the anterior one-third of the vertebral body depicts a “watershed” area of blood supply in which a minor fracture could lead to vascular disruption and subsequent ONV. Based on this presumption, some researchers proposed intervertebral autogenous bone grafting, which is similar to nonunion therapy in the lower or upper extremities. However, the 5-year follow-up results are still unsatisfactory because of the high nonunion rate and recollapse of the injured vertebra [24].

Augmentation of the vertebra combined with SSPI has multiple benefits in dealing with ONV. The penetration of cement in the vertebral cleft could redistribute stress. Meanwhile, the cauterization effect of the cement on the peripheral nerve distributing near the cleft could produce an excellent analgesic effect. SSPI alters the stress transmission in the spinal vertebra, which could reduce inflammation in the vertebral body and eventually lead to pain relief. In some short-term studies, the authors have recommended long segment instrumentation, including two vertebrae above and below the damaged vertebra [8, 16]. They also emphasized massive cement injection to promote biomechanical stability. Nevertheless, extended fixation could lead to more severe damage to the soft tissue, which could in turn influence segment stability. Meanwhile, according to biomechanical research on the cement injection technique, the pull-out strength could be magnified 1.7 times after the pedicle screw was augmented with PMMA cement. The morphology of the cement is more critical than cement volume in treating ONV in determining the long-term clinical effect. Based on the above viewpoints, we adopted SSPI using a cement pedicle to stabilize the injured segment. Cement volume was precisely decided by the morphology of the distribution of cement. Due to the high risk of refracture in the caudal area adjacent to the cleft [14, 21], we adopted double tunnels to augment the cancellous bone beneath the cleft.

Theoretically, this method can generate excellent treatment effects. However, in spite of these optimal methods, the long-term results were disappointing. Improvements in symptoms and radiographic imaging were maintained for 6–12 months. We compared the data at the final follow-up visit with the preoperative data and we found no statistically significant difference in VAS, ODI, kyphotic angle, and vertebral height. The kyphotic angle increased in 26 patients with deteriorative LBP.

We speculate that the negative results are due to multiple factors. First, cement penetration through the interface is unsatisfactory because local osteosclerosis is a common phenomenon in ONV. The injection of PMMA cement into a cystic cavity is thought to achieve substantially less interdigitation with the nearby bone than injection into a mostly intact trabecular bone [8, 15, 17, 25]. Second, in spite of the excellent short-term stability SSPI could offer, cement injection can eliminate the possibility of bone union in the cleft, which means that permanent anterior column reconstruction could hardly be achieved. The instrumentation might fail because of the deteriorative osteoporosis over a long period of time. Third, in our study, we observed significant vertebral body height loss at the caudal vertebra in 23 patients. The exact reasons for this phenomenon are hard to confirm. However, the difference in stiffness coefficient between PMMA cement and trabecular bone might lead to a fracture at the cement–bone interface, contributing to the postoperative increase in kyphotic angle.

There were several limitations in our study. First, because of the complicated pathology of ONV, patients in different pathologic stages may have various reactions to the same treatment. Bias could not be avoided in this uncontrolled study. Second, the sample size was relatively small, which may limit its statistical power. Third, during cement augmentation, the cement volume, which was decided by the morphology of cement distribution, varied within a wide range. This may have a potential influence on the final results.

## Conclusion

Our outcomes indicate that in cases of ONV, TCA with SSPI cannot efficiently offer long-term stability and prolonged relief of back pain throughout a 5-year follow-up period. Patients may experience short-term symptomatic relief, but over time, treatment effects and radiologic improvement are gradually lost. We also noted ongoing kyphotic alteration of the vertebral body throughout the follow-up period in the majority of the patients. Therefore, we do not recommend SSPI concurrently with TCA for treating ONV.

## Data Availability

The datasets used and/or analyzed during the current study are available from the corresponding author on reasonable request.

## Abbreviations

TCA: Transpedicular intracorporeal cement augmentation
SSPI: short segmental posterior instrumentation
VAS: visual analogue scale score
ODI: Oswestry Disability Index
ONV: Osteonecrosis of the vertebral body
MRI: magnetic resonance imaging
OVCF: osteoporotic vertebral compression fractures
LBP: low back pain
CT: computed tomography
PMMA: polymethylmethacrylate
PACS: picture archiving and communication system
PKP: percutaneous kyphoplasty
PVP: percutaneous vertebroplasty
